# 2232: Acute kidney injury in patients on SGLT-2 inhibitors: A propensity matched analysis

**DOI:** 10.1017/cts.2017.100

**Published:** 2018-05-10

**Authors:** Rocco Ferrandino, Girish Nadkarni, Priti Poojary, Aparna Saha, Bart Ferket, Kinsuk Chauhan, Steven Coca

**Affiliations:** Icahn School of Medicine at Mount Sinai, New York, NY, USA

## Abstract

OBJECTIVES/SPECIFIC AIMS: In June 2016, the FDA cautioned against the use of SGLT-2 inhibitors because of increased risk of acute kidney injury (AKI) after 101 cases of AKI were reported between March 2013 and October 2015. This study seeks to determine risk of AKI associated with SGLT-2 inhibitors in a large cohort of type 2 diabetic patients. METHODS/STUDY POPULATION: Retrospective cohort study including SGLT-2 inhibitor users and nonusers in the Mount Sinai Chronic Kidney Disease Registry between January 2013 and September 2016. SGLT-2 inhibitor users and nonusers were type 2 diabetics with new SGLT-2 inhibitor prescription after January 2013 and an outpatient visit between 2013 and 2015, respectively. Subjects were propensity matched by nearest neighbor method based on demographics, comorbidities, laboratory values, medications, estimated glomerular filtration rate (eGFR), and length of follow-up. The primary end point was AKI (defined by KDIGO laboratory algorithm) occurring during the follow-up period. RESULTS/ANTICIPATED RESULTS: In total, 372 SGLT-2 inhibitor users [mean age 63 years; 205 (55%) men] and 372 [mean age 63; 194 (52%) men] nonusers were included in the primary analysis. Proportions of AKI events defined by KDIGO criteria in users and nonusers were 4.0% and 10.0%, respectively. Adjusted odds ratio for AKI was 1.00 (95% CI, 0.28–2.62). Median peak serum creatinine measurements during AKI events for user and nonuser groups were 1.60 (IQR 1.36–1.78) and 1.88 (IQR 1.55–2.44) (*p*=0.02), respectively. Sensitivity analyses yielded similar results. DISCUSSION/SIGNIFICANCE OF IMPACT: These findings suggest that there is no evidence of increased odds of AKI in SGLT-2 inhibitor users compared with propensity-matched nonusers with type 2 diabetes.

